# Hearing loss and brain disorders: A review of multiple pathologies

**DOI:** 10.1515/med-2021-0402

**Published:** 2021-12-15

**Authors:** Oluwafemi Gabriel Oluwole, Kili James, Abdoulaye Yalcouye, Ambroise Wonkam

**Affiliations:** Division of Human Genetics, Faculty of Health Sciences, University of Cape Town, 3.14 Wernher & Beit North Building, P.O Box 7925, Cape Town, South Africa

**Keywords:** hearing loss, brain disorders, pathology, correlated risk factors

## Abstract

Several causative factors are associated with hearing loss (HL) and brain disorders. However, there are many unidentified disease modifiers in these conditions. Our study summarised the most common brain disorders associated with HL and highlighted mechanisms of pathologies. We searched the literature for published articles on HL and brain disorders. Alzheimer’s disease/dementia, Parkinson’s disease, cognitive impairment, autism spectrum disorder, ataxia, epilepsy, stroke, and hypoxic-ischaemic encephalopathy majorly co-interact with HL. The estimated incidence rate was 113 per 10,000 person-years. Genetic, epigenetic, early life/neonatal stress, hypoxia, inflammation, nitric oxide infiltration, endoplasmic reticulum stress, and excess glutamate were the distinguished modifiers identified. Various mechanisms like adhesion molecules, transport proteins, hair cell apoptosis, and neurodegeneration have been implicated in these conditions and are serving as potential targets for therapies. To improve the quality of life of patients, these understandings will improve clinical diagnoses and management of HL and brain disorders.

## 1 Introduction

Hearing loss (HL), deafness, or hearing impairment can be described as a total or partial inability to hear sounds. HL could be conductive, sensorineural, or mixed, depending on the parts of the auditory system being affected [1]. Globally, approximately 400 million people are diagnosed of HL [2,3] and millions of people suffered from one form of brain disorders. Environmental and genetic factors have been identified as the leading contributors. Syndromic HL is associated with numerous syndromes like Waardenburg syndrome, branchiootorenal syndrome, Usher syndrome, Pendred syndrome, keratitis-ichthyosis-deafness syndrome, and Alport syndrome [1,4]. Non-syndromic HL is a form of sensorineural HL that is not linked to a syndrome [5].

HL can cause several neurological and psychiatric complications that can reduce quality of life [6]. Some of the complications may include cognitive impairment, depression, dementia, and comorbidities like cardiovascular diseases and diabetes [4,7,8]. More so, the feelings of irritability, anxiety, and rage in patients with HL may emanate from undiagnosed brain disorders [2].

Physiologically, the brain is the true organ of hearing because the brain processes the sounds that are transmitted from the ears. Hearing is a complex process that requires connection between the peripheral and central auditory processing systems [9], this connectivity enables the sharing of aetiological factors. Impaired cochlear blood perfusion and microvascular damage in the ear can cause sensorineural HL, as well as certain brain disorders. This review aims to identify commonly associated brain disorders with sensorineural HL and explains the plausible mechanisms by which HL co-interacts with the brain disorders. The understanding of these multiple pathologies will contribute to the clinical management of patients.

## 2 Methods

### 2.1 Search method

We searched five electronic databases “PubMed, HubMed, Google Scholar, MEDLINE, and Embase” for published articles on HL and brain disorders between the years 2000 and 2021.

We used a combination of strings and MeSH terms (hearing loss) AND with OR (hearing impairment) AND with OR (deafness) AND with OR (brain) AND with OR (brain disorders) AND with OR (brain disease). The strings were adapted to each database entries.

#### 2.1.1 Selection criteria

We prioritized the articles that have the searched terms in their title or abstract. Additionally, the articles that reported clinical, scientific, or technological findings on HL with brain disorders in humans were selected. The articles considered relevant had the following objectives:

1. Reported the incidence or clinical diagnoses of HL with brain disorders.
2. Evaluated the causative factors of HL with brain disorders.
3. Investigated novel potential targets for therapy in HL with brain disorders.

#### 2.1.2 Extraction and data presentation

The full texts of the selected articles were downloaded to Zotero electronic reference manager for reference. We retrieved meta-data like demographics, the number of study participants and cases. We estimated the incidence rates as a measure of new cases/total, that is, the number of study participants considered at risk. The findings were summarized in Table 1. We provided a diagrammatic illustration to explain the underlying multiple pathologies and the mechanisms of HL co-interaction with brain disorder by using the biorender app (https://app.biorender.com/).

**Table 1:** Studies on brain disorders and hearing loss

Type of diseases	Methods	Major findings	Total number of cases	References
Alzheimer's disease (AD) and dementia	Studies that performed PTA, ABR, and central auditory processing tests on participants diagnosed with dementia/AD. Patients were compared with control groups	There was a significant association between central auditory dysfunction and dementia/AD in the primary auditory cortex with evidence of abnormal blood glucose levels	1,045	[10,11,12,13,14]
	Studies that performed longitudinal research on patients diagnosed with sensorineural HL and investigated association with dementia in participants	During the average 5 year follow-up period, the incidence rate of dementia in the sensorineural hearing loss (SNHL) cohort was 6.5 per 1,000 person-years compared with 5.09 per 10,000 person-years in the comparison group. HL was independently associated with a high incident rate of dementia in mild, moderate, and severe HL	17,523	[15,16,17]
Parkinson's disease (PD)	Assessments of HL were evaluated in PD patients with audiometric testing, and a battery of central auditory processing tests	Compared to the control group, PD patients reported greater HL	184	[18,19,20,21,22,23,24]
Cognitive impairment	Patients diagnosed with HL were recruited for the study and completed the modified Mini-Mental State Examination, cognitive test, and linear mixed models for correlation	About one-sixth (15.7%) of the patients studied had cognitive decline; 10.1% had functional decline among individuals with HL. There was also an association with history of stroke	5,445	[17,25,26,27]
Autism spectrum disorder (ASD)	Audiological evaluation was performed using PTA tests in children diagnosed with autism	Mild-to-moderate HL was diagnosed in 7.9% and unilateral HL in 1.6%, profound bilateral HL was diagnosed in 3.5% of all cases	199	[28]
Epilepsy and ataxia	A retrospective review of the detailed neurological and neuroradiological features were performed in nine children	All children presented with tonic–clonic seizures in infancy. Later, with non-progressive, cerebellar ataxia and profound HL	9 children, 1 adult	[29,30]
Hypoxic ischaemic encephalopathy (HLE)	Dual-stage hearing screening tests, including automated otoacoustic emissions and ABR tests, were performed in new-borns suspected to have developed HL	The study affirms a significant association between HL in term infants who have moderate/severe HLE with evidence of abnormal blood glucose and multi-organ dysfunction (*p* = 0.006)	42	[31]
Stroke	Neurological and general examinations were performed. Followed by audiogram and MRI screening	Acute stress was recognised and moderate sensorineural HL with the presence of bilateral temporal ischemic stroke lesions	1	[32]

## 3 Results

**Figure 1:**
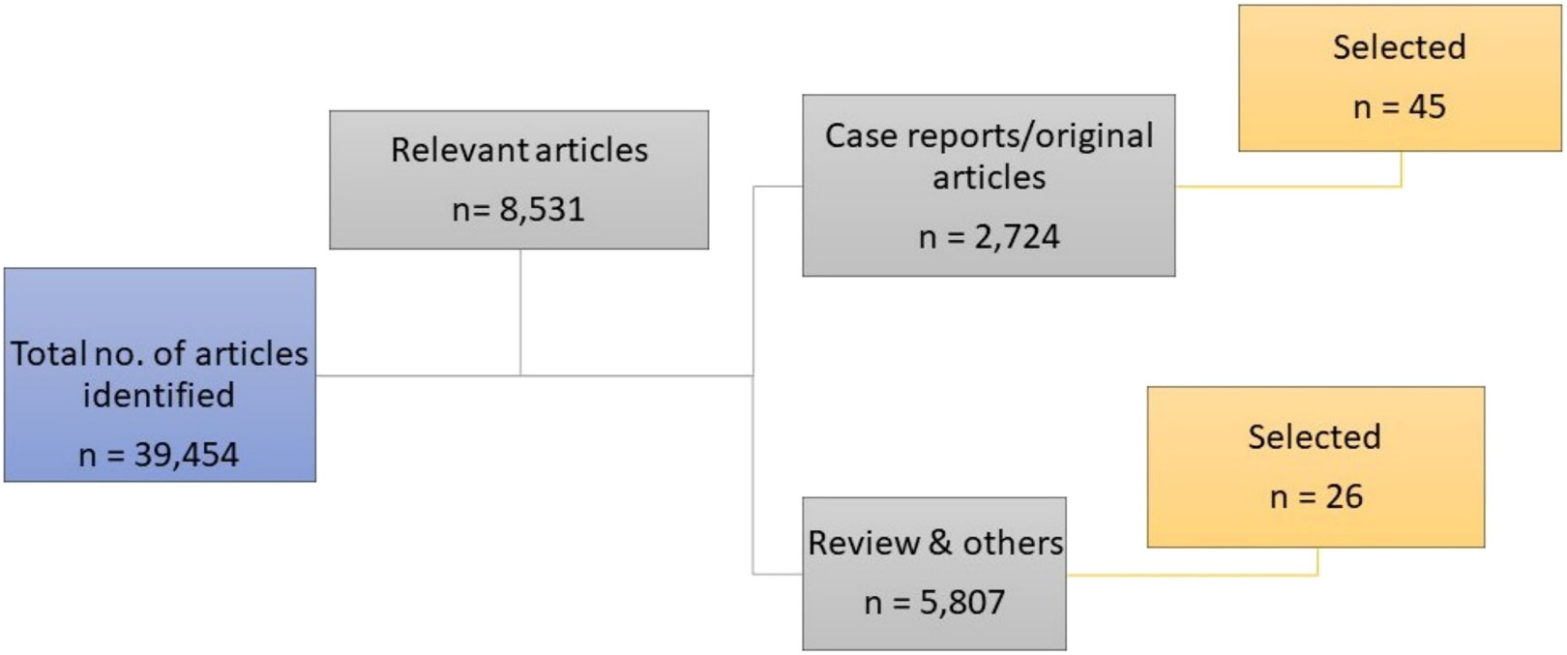
Filtering processes and article selections in the study.

The search engines gave an output of 39,454 articles, of which 8,531 articles were initially considered as relevant articles. They include case reports, reviews, short communications, letters to the editor, and original articles. However, after the filtering processes by using stringent criteria to select case reports, reviews, and original articles that presented meta-data or detailed information on the topic of HL with brain disorder only, we identified 71 important articles, of which 45 articles were original articles (Figure 1). The focus of the 45 articles was on molecular pathology, diagnosis, incidence, or genetics.

The results of our analyses showed that Alzheimer’s disease/dementia, Parkinson’s disease (PD), cognitive impairment, autism spectrum disorder, ataxia, epilepsy, hypoxic-ischaemic encephalopathy, and stroke have been reported to co-interact with HL (Table 1). The overall number of cases was 2,765 of 24,447 participants, and the estimated incidence rate was 0.113 per person-year or 113 per 10,000 person-years.

**Figure 2:**
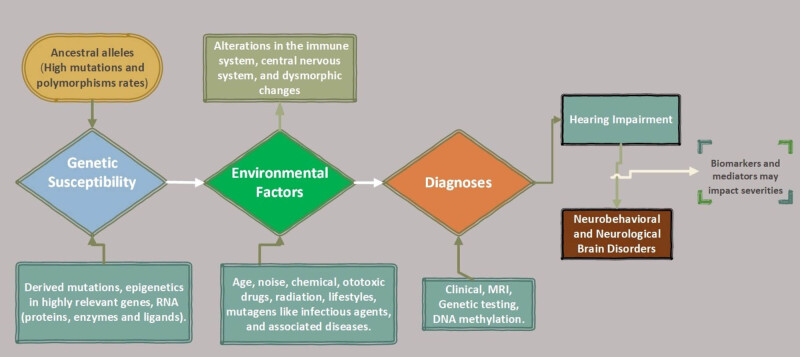
The description of intrinsic, extrinsic factors and disease modifiers underlying the pathologies of HL co-interactions with brain disorders.

The demographic data derived from the studies imply that adult patients usually have Alzheimer’s disease, Parkinson’s disease, and cognitive impairment, whereas the infants and children had autism spectrum disorder (ASD), attention deficit hyperactivity disorder, and cerebral palsy with HL. Geographically, the studies were performed mainly in Europe and Asia. The researchers used different methods for diagnoses (Table 1), but generally the clinical presentations of the patients were similar. Likewise, the findings signified those complex mechanisms such as hereditary, acquired, or combination of factors including environmental and physical abnormalities in the causes of HL and brain disorders (Figure 2).

**Figure 3:**
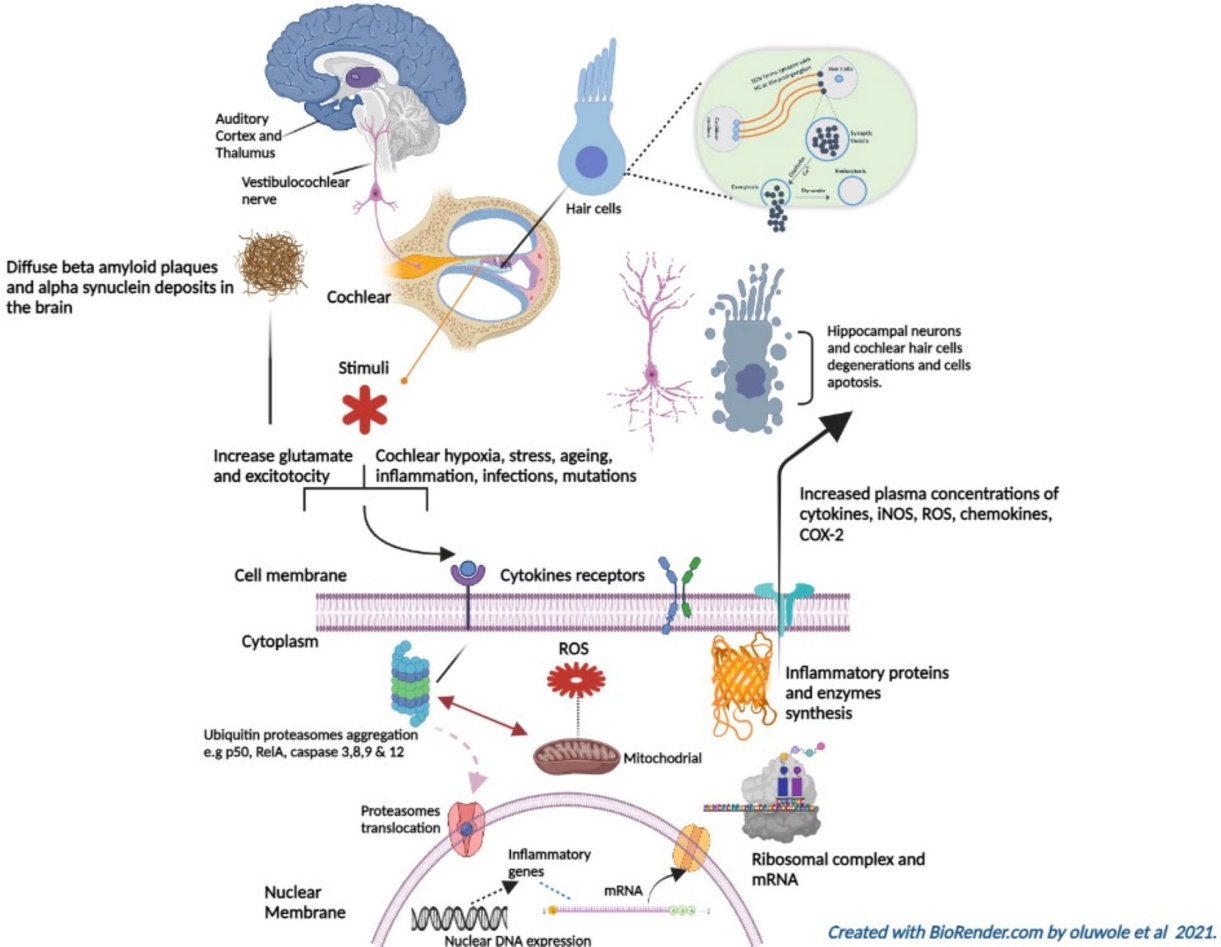
A description of auditory signal pathway and possible insults to the auditory nervous system. The auditory nervous pathway originates from spiral ganglion cells in the cochlea. The pathological changes that occur in the cochlear, like inflammation can initiate peripheral immune responses and activations of inflammatory genes as well as over production of reactive oxygen species (ROS) and other inflammatory biomarkers that can affect the mitochondrial and diffuses to the central nervous system to cause neurodegeneration and cell apoptosis. Also, mtDNA mutations can cause mitochondrial dysfunction and cell apoptosis.

Furthermore, ten studies distinguished the roles of methylated genes and reported single nucleotide polymorphisms (SNPs), that overlap between HL and brain disorders (Table 2). We summarised, in Figure 3, the mechanisms involving the multiple pathologies of HL with brain disorders such as stress, inflammation, genetic factors, and environmental factors such as heavy metals, pesticides, bisphenol A; polybrominated diphenyl ethers; polychlorinated biphenyls; perfluorocarboxylic acids; and perfluorooctanesulfonate.

**Table 2:** Epigenetic studies involving HL and brain disorders genes

Methylated genes	Brain disorders	Proposed mechanisms	Associated SNPs/mutations	References
*DNMT1*	Autism, cerebellar ataxia, Huntington disease	DNMT1 negatively impacts retrograde trafficking and autophagy	rs10418707, rs10423341, rs2114724, and rs759920	[33,34,35]
*NSD1*	AD, and intellectual disability syndrome	Most of the mutations identified are predicted to disrupt the reading frame in a way that causes early translational termination and/or activates non-sense-mediated decay	Multiple mutations have been reported including the c.2314delG, c.2362C > T, c.5296C > A	[36–38]
*EZH2*	Ataxia	Enhanced nuclear H3K27me3 affects cell cycle and neuronal survival through reverse transcription mechanisms and complexes	Ser652 and Ser734 sites methylation	[39]
*EHMT1*	Intellectual disability, schizophrenia, and psychosis, autism, PD, and HD	The addition of methyl groups to histone H3 lysine 9 linked to genomic imprinting, X-inactivation, and heterochromatin formation	Multiple H3K9 dimethylation has been reported	[40]
*KMT2D*	Kabuki syndrome (intellectual disability) and multiple malformations syndrome	Methylation leads to the truncation of C-terminal SET catalytic domain, likely resulting in the loss of enzymatic function	Multiple mutations have been reported including the p.Cys1430Arg and p.Cys1471Tyr	[41–43]
*CHD7*	ASD and sleep disorder	Mechanisms not fully understood but were thought to control glia activation and causes hyperserotonemia	Multiple mutations have been reported in non-human subjects	[44]

## 4 Discussion

Complex biological pathways and modifiers are involved in the onset of HL and brain disorders. The anatomical structures of the cochlea of the ear, and the brain show that the cochlea consists of hair cells that allow the neural conduction with the brain by the auditory nerves at the synapses [1,9,45,46,47,48,49]. The damage(s) to the auditory nerves, cortex, or thalamus is/are capable of inducing HL and brain disorders. We identified studies that showed that a bilateral lesion of the superior temporal gyrus and the thalamic nuclei of the brain caused HL, motor, sensory, and neuropsychological impairments [50,51]. Also, a haemorrhagic lesion in the right thalamus due to an aneurysm was linked to the aetiology of bilateral HL in children [52,53]. More so, cerebral palsy co-interacts with childhood HL [54].

The damages to the thalamus and the central auditory system can be caused by a stroke, head injury, brain tumours, or neurodegeneration [52,53]. These damages may lead to loss of auditory cortex, and may contribute to the down regulation of neural activities in the brain [55].

In essence, our study advocate for the use of advanced technology such as magnetic resonance imaging (MRI) in the diagnosis and early detection of HL and brain disorders.

Furthermore, stress and inflammation contribute to the onset of HL and brain disorders. Stress has multiple effects that can be linked to the release of adrenaline and vasoconstrictors that may narrow blood flow into the tissues of inner ear [56]. Studies have shown a strong correlation (∼32% of 9,756 participants) between HL and different types of daily stressors like occupational, poverty, long-term illness, lack of sleep, and higher burnout [57]. Also, the responses to stress via the hypothalamic-pituitary-adrenal (HPA) axis have been implicated in HL and various brain diseases [58]. The pathology may be linked to the release of cortisol and an imbalance of the *N*-methyl-d-aspartate and α-amino-3-hydroxy-5-methyl-4-isoxazolepropionic acid receptors in the cochlea that can cause excess glutamate and glycine release, followed by excitotoxicity, and altered gene expression in the cochlea and the brain as described in Figure 2.

Stress can induce inflammation or hypoxia in the hair cell by affecting the epithelial cells in the brain to induce immune responses and cellular infiltration [46]. Stress-induced inflammation or hypoxia in the cochlea has been shown to be modulated by the macrophages [58]. Besides, virus-triggered inflammation of the meninges with subsequent spread to the cochlea could initiate the pathological processes that include cytokines (interleukin 1-beta [IL-1beta], IL-6,), overproduction of ROS, inducible nitric oxide synthase (iNOS), and tumour necrosis factor-alpha. The cytokines are known to enter the brain and cause neurodegeneration in the cortex and the thalamus to impair auditory processes [59,60,61]. And the iNOS has been linked to the cochlear pathology through blood flow to the middle ear, outer hair cell, and vestibular functions in sensorineural HL [62,63], as well as brain diseases like parkinson’s disease (PD), alzheimer’s disease (AD), amyotrophic lateral sclerosis (ALS), huntington's disease (HD), autism spectrum disorder (ASD), and stroke [64].

Furthermore, there is genetic overlap between HL and AD pathology [65]. Studies have shown that genetic, environmental, and birth-related issues contribute largely to HL [66,67]. The environmental and genetic information of an individual can pose differences in the way diseases like HL and brain disorders co-interact. Mutated genes in HL share biological pathways with genes that regulate adhesion, transport, and synapses proteins peripherally and centrally [68], which have been implicated in the biological functions like adhesion, intracellular transport, neurotransmitter release, ionic homeostasis, and cytoskeletons. To date, mutations in over 150 genes have been linked to HL (http://hereditaryhearingloss.org/). The *GJB2* and *GJB6* genes are the most common in HL [69]. Perhaps, mutations in a single gene or combination of genes can result in the onset of the multiple pathologies in HL and brain disorders. Unlike the European and American populations that have been majorly investigated, research needs to focus more on the under-represented populations because they are known for genetic diversities that could play some important ramifications for diseases and treatment [70].

Lastly, the epigenetic factors contribute to the aetiology of HL and brain disorders. Epigenetic modifications and regulatory mechanisms are important biological events that switch on or off genes for hearing and brain functions. More than one hundred microRNAs are regulated by epigenetic mechanisms, and about 50% of them are modulated by DNA methylation and have been associated with different diseases [71]. Importantly, epigenetic factors can influence the transcriptomics during the developmental stage. Of which, the gene expression in the thalamus has been described to be susceptible to epigenetic changes [72]. Currently, resources like umgear (www.umgear.org) document epigenetic changes that influence gene expressions for further studies in this area.

In conclusion, there are limitations in our study which include that we only reviewed articles published in English between the years 2020 and 2021. Also, we analysed articles that have free full text. Nonetheless, the significance of our study is in the identification of various brain disorders that are co-interacting with HL, and the raw incidence rate to expect per population. Our study gave an overview of diseases that may present clinically with HL. We emphasised genetics, epigenetic, damaged cortical/thalamus, stress, inflammation, and immune system dysfunction as primary contributors to the onset of HL and brain disorders. The identification of the multiple pathologies strengthen research ideas in this area and will promote healthcare awareness, contribute to target discoveries and novel treatments, as well as improve the diagnosis and care for people affected with HL and brain disorders.

## Abbreviations

ASD: autism spectrum disorders
HL: hearing loss
HPA-axis: hypothalamic-pituitary-adrenal axis
HLE: hypoxic ischaemic encephalopathy
IHC: inner hair cells
MRI: magnetic resonance imaging
NO: nitric oxide
NMDA: *N*-methyl-d-aspartate
NSHL: non-syndromic hearing loss
SGNs: spiral ganglion neurons
AMPA: α-amino-3-hydroxy-5-methyl-4-isoxazolepropionic acid
